# Comparison of Clinical Efficacy and Safety between Indacaterol and Tiotropium in COPD: Meta-Analysis of Randomized Controlled Trials

**DOI:** 10.1371/journal.pone.0119948

**Published:** 2015-03-23

**Authors:** Jung Soo Kim, Jinkyeong Park, Seong Yong Lim, Yeon-Mok Oh, Kwang Ha Yoo, Yong Bum Park, Seung Soo Sheen, Min-Ji Kim, K. C. Carriere, Ji Ye Jung, Hye Yun Park

**Affiliations:** 1 Division of Pulmonary and Critical Care Medicine, Department of Medicine, Samsung Medical Center, Sungkyunkwan University School of Medicine, Seoul, Korea; 2 Department of Internal Medicine, Uijeongbu St. Mary's Hospital, The Catholic University of Korea College of Medicine, Uijeongbu, Korea; 3 Department of Medicine, Kangbuk Samsung Hospital, Sungkyunkwan University School of Medicine, Seoul, Korea; 4 Department of Pulmonary and Critical Care Medicine, Clinical Research Center for Chronic Obstructive Airway Diseases, Asan Medical Center, University of Ulsan College of Medicine, Seoul, Korea; 5 Department of Internal Medicine, Konkuk University College of Medicine, Seoul, Korea; 6 Department of Pulmonary, Allergy and Critical Care Medicine, Hallym University Medical Center, Hallym University Kangdong Sacred Heart Hospital, Seoul, Korea; 7 Department of Pulmonary and Critical Care Medicine, Ajou University School of Medicine, Yeongtong-gu, Suwon, Korea; 8 Biostatistics team, Samsung Biomedial Research Institute, Seoul, Korea; 9 Department of Mathematical and Statistical Sciences, University of Alberta, Edmonton, Alberta, Canada and Research Professor, Biostatistics and Clinical Epidemiology Center, Samsung Medical Center, Seoul, Korea; 10 Division of Pulmonology, Department of Internal Medicine, Institute of Chest Disease, Yonsei University College of Medicine, Seoul, Korea; Bascom Palmer Eye Institute, University of Miami School of Medicine, UNITED STATES

## Abstract

Two once-daily inhaled bronchodilators, indacaterol and tiotropium, are widely used as first-line therapy in stable COPD patients. This study was performed to compare the clinical efficacy and safety between indacaterol and tiotropium in patients with moderate-to-severe COPD. MEDLINE, EMBASE, and Cochrane Central Register of Controlled Trials were searched to identify all published randomized controlled trials (RCTs). The primary outcome was trough forced expiratory volume in 1 second (FEV_1_) at week 12. Four RCTs were eligible for inclusion (three RCTs with moderate-to-severe COPD patients and one RCT with only severe COPD patients). Trough FEV_1_ at weeks 12 and 26 were not significantly different between indacaterol and tiotropium by the standardized mean difference with 0.014 (95% CI, -0.036, 0.063, *I^2^*= 23.5%) and with 0.037 (95% CI, -0.059 to 0.133, *I^2^*= 0%) along with differences in means of 0.003L and 0.014L, respectively. Indacaterol and tiotropium also showed similar St. George`s Respiratory Questionnaire (SGRQ) total scores and percentages of patients with SGRQ improvement (≥ 4 units) at week 26. The incidences of nasopharyngitis, serious cardiovascular events, and serious adverse events were not different between indacaterol and tiotropium, while those of cough (OR = 1.68, *P* < 0.001, and RR = 1.63) and COPD worsening (OR = 1.18, *P* = 0.003, and RR = 1.12) were higher for indacaterol than tiotropium. However, when one study with only severe COPD patients was removed from the meta-analysis, the difference in the incidence of COPD worsening between indacaterol and tiotropium became non-significant (OR = 1.13, *P* = 0.204, and RR = 1.09). The clinical efficacy and serious adverse events between indacaterol and tiotropium were equivocal in patients with moderate-to-severe COPD. Cough is a common complaint associated with indacaterol, and COPD worsening needs to be carefully monitored in severe COPD patients when treated with indacaterol.

## Introduction

Chronic obstructive pulmonary disease (COPD) is a major global health burden with an estimated prevalence of 300–600 million adults [1], and almost three million deaths annually [2, 3]. It is characterized by progressive and irreversible airflow limitation, which adversely affect respiratory symptoms, exercise tolerance, and quality of life [4]. Bronchodilators play a key role in palliation of symptoms in patients with COPD, and long-acting bronchodilators are currently recommended as maintenance bronchodilator therapy [4]. In particular, once-daily inhaled bronchodilators provide 24-h therapeutic action, which leads to improved adherence and efficacy compared with short-acting or twice-daily inhaled bronchodilators [5–8].

Various once-daily inhaled bronchodilators have been introduced. Among them, once-daily anticholinergic tiotropium and once-daily β_2_-agonist indacaterol have been widely used as maintenance treatment in cases of stable COPD. Both tiotropium and indacaterol have shown significant clinical benefits in terms of improving lung function, symptoms, and quality of life over placebo, and they have no more safety concerns than the placebo have in stable patients with moderate to severe COPD [9–12]. The concurrent use of an inhaled long-acting β_2_-agonist (LABA) and inhaled long-acting muscarinic antagonist (LAMA) provides superior efficacy with no increase in clinically relevant adverse events compared with individual agents [13], but current guidelines recommend the combined use of LABA and LAMA when symptoms are not improved by a single agent [4]. Thus, single-agent therapy with LABA or LAMA remains the initial treatment of choice in symptomatic COPD patients [4]. As there is lack of evidence for recommending one class of long-acting bronchodilators over another for initial treatment, the present study was performed to systematically compare current reports on the clinical efficacy and safety of indacaterol versus tiotropium in stable patients with moderate to severe COPD.

## Methods

### Literature search

We identified published studies from MEDLINE, EMBASE, and the Cochrane Central Register of Controlled Trials (to July 1, 2014) databases using keywords related to COPD, indacaterol and RCTs (*see* S1 Table for details). The search filters provided by SIGN (http://www.sign.ac.uk/methodology/filters.html) were used. The search was without language restriction and included unpublished studies. Trials published solely in abstract form were excluded because the methods and results could not be fully analyzed.

### Selection criteria

To meet our specific inclusion criteria, each study was required to satisfy the followings: (1) patients with stable moderate to severe COPD according to Global Initiative for Chronic Obstructive Lung Disease (GOLD) diagnostic criteria [4]; (2) randomized control trials (RCTs) with comparison of inhaled indacaterol vs. tiotropium; (3) at least 12 weeks of follow-up; (4) report outcome of trough forced expiratory volume in 1 second (FEV_1_) at week 12; (5) written in English.

### Data extraction and risk of bias assessment

The title and abstract were independently analyzed by three authors (H.Y.P, J.Y.J, and J.S.K) for screening. They independently assessed all studies for inclusion based on the criteria for study design, outcome and intervention for participants. After they obtained full texts that could be potential candidates, they assessed and confirmed eligibility for the analysis. Disagreements were discussed and resolved by consensus. The two reviewers assessed the risk of bias of included studies for sequence generation, allocation concealment, blinding of participants and researchers, blinding of outcome assessment, incomplete outcome data addressed, and free of selective reporting as recommended in the Cochrane Handbook of Systematic Reviews 5.1. [14]. The authors compared their evaluations and reassessed the studies together as necessary. Disagreement was solved by discussion and consensus between the authors.

### Primary and secondary outcome analysis

The primary outcome was comparison of trough (24-h postdose) FEV_1_ of indacaterol with tiotropium at week 12 following treatment. Secondary outcomes consisted of comparison of trough FEV_1_, St. George`s Respiratory Questionnaire (SGRQ) total score and the minimal clinically important difference (MCID) of SGRQ total score at week 26. Based on empirical data and interviews with patients, a mean change score of 4 units was associated with slightly efficacious treatment, which is referred to as MCID [15].

We also assessed detailed adverse events, including the incidence of any adverse events, nasopharyngitis, cough, COPD worsening, serious adverse events, and serious cardiovascular events (cardiac failure and myocardial ischemic disease).

### Statistical analysis

We used Comprehensive Meta-Analysis, Version 2 (Englewood, NJ, USA; Biostat, Inc.), to carry out meta-analysis of the included studies. Outcomes were pooled using standardized differences in means (SMD) under the fixed effects model or odds ratio (OR). We also added difference in means (MD) and relative ratios (RR) whenever possible. The precision of the estimates was quantified by the 95% confidence interval (CI). Heterogeneity was measured by the Higgins and Green *I* ^*2*^ test, which is calculated as *100%×(Q*–*df)/Q*, where *Q* is the observed chi-squared statistic and the degrees of freedom (df) is the number of studies less one [14]. The *I* ^*2*^ ranges between 0% (no heterogeneity) and 100% (maximal heterogeneity), and heterogeneity was considered to be substantial at *P* < 0.10 and *I* ^*2*^ > 50% [14]. Heterogeneity was explored with sensitivity analysis. We also conducted the potential publication bias with Egger’s regression test and the funnel-plot based Trim and Fill method [16]. *P* values < 0.05 (two-tailed test) was considered significant. The methodological quality of the selected trials was assessed using the criteria described in the Cochrane Handbook [17].

## Results

**Fig 1:**
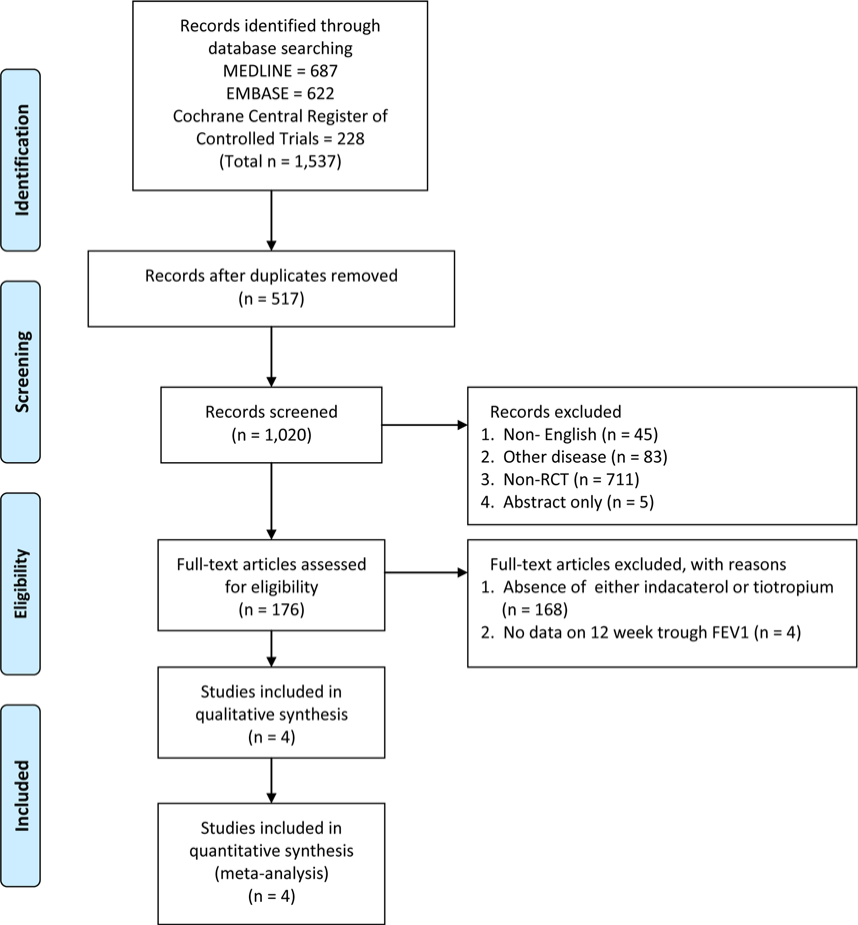
Flowchart for identification of studies used. RCT = randomized controlled trial.

Fig. 1 shows how relevant studies were identified. A total of 1,537 articles were found by searching databases. We excluded 517 duplicated articles and an additional 844 articles based on our inclusion criteria. One hundred seventy two trials were excluded as 168 trials was absent of either indacaterol or tiotropium and four did not provide details of trough FEV_1_ at week 12. Thus, four randomized controlled studies finally met the inclusion criteria [18–21]. A total of 6,819 subjects were enrolled with 3,407 in the indacaterol 150 μg group and 3,412 subjects in the tiotropium 18 μg group. The mean age of patients was 63.7 years, and 73% were male. Three studies (INHANCE, INTENSITY, and SHINE) had entry criteria for symptomatic patients with moderate to severe COPD [18–20], while one study (INVIGORATE) included only severe COPD patients [21]. Three studies (INHANCE, INTENSITY, and INVIGORATE) compared clinical outcomes between indacaterol 150 μg and tiotropium 18 μg. In the one remaining study (SHINE), patients received QVA149 (indacaterol/glycopyrronium 110/50 μg), indacaterol 150 μg, glycopyrronium 50 μg, open label tiotropium 18 μg, or placebo to investigate efficacy and safety. The duration of follow-up ranged from 12 weeks to 52 weeks. Table 1 presents a summary of the general characteristics of the four RCTs [18–21].

**Table 1:** Characteristics of included studies.

Study	Treatment duration (weeks)	COPD criteria (GOLD)	Number of subjects	Men,%	Age (mean)	Drug and Dose	Baseline FEV_1_ L (% Predicted)	Primary outcome
**Donohue et al 2010 [18] (INHANCE study)**	26	Moderate to Severe	416	62	63.4	Indacaterol 150μg	1.52 (56.1)	Trough FEV1 at week 12
			415	65	64.0	Tiotropium 18μg	1.45 (53.9)	
**Buhl et al 2011 [19] (INTENSITY study)**	12	Moderate to Severe	794	70	63.6	Indacaterol 150μg	1.53 (54.6)	Trough FEV1 at week 12
			799	67	63.4	Tiotropium 18μg	1.52 (54.3)	
**Bateman et al 2013 [20] (SHINE study)**	26	Moderate to Severe	476	74	63.6	Indacaterol 150μg	1.5 (54.9)	Trough FEV1 at week 26
			480	75	63.5	Tiotropium 18μg	1.5 (55.1)	
**Decramer et al 2013 [21] (INVIGORATE study)**	52	Severe	1721	78	64.0	Indacaterol 150μg	1.13 (40.2)	Trough FEV1 at week 12
			1718	76	64.0	Tiotropium 18μg	1.14 (40.7)	

COPD = Chronic obstructive pulmonary disease; FEV_1_ = forced expiratory volume in 1 second; GOLD = Global Initiative for Chronic Obstructive Lung Disease.

### Risk of bias in the included studies

The assessments performed by the authors of each risk of bias item for each included RCT are summarized in Table 2. A high risk of bias for blinding of participants was reported in two studies due to open labeled study.

**Table 2:** Risk of bias amongst included studies.

Source	Sequence generation	Allocation concealment	Blinding of participants and researchers	Blinding of outcome assessment	Incomplete outcome data addressed	Free of selective reporting
**Donohue et al 2010 [18] (INHANCE study)**	Unclear	Low risk	High risk	Low risk	High risk	Low risk
**Buhl et al 2011 [19] (INTENSITY study)**	Unclear	Low risk	Low risk	Low risk	Low risk	Low risk
**Bateman et al 2013 [20] (SHINE study)**	Unclear	Unclear	High risk	Low risk	Low risk	Low risk
**Decramer et al 2013 [21] (INVIGORATE study)**	Low risk	Low risk	Low risk	Low risk	Low risk	Low risk

COPD = Chronic obstructive pulmonary disease; OR = Odds ratio.

### Primary outcome

**Fig 2:**
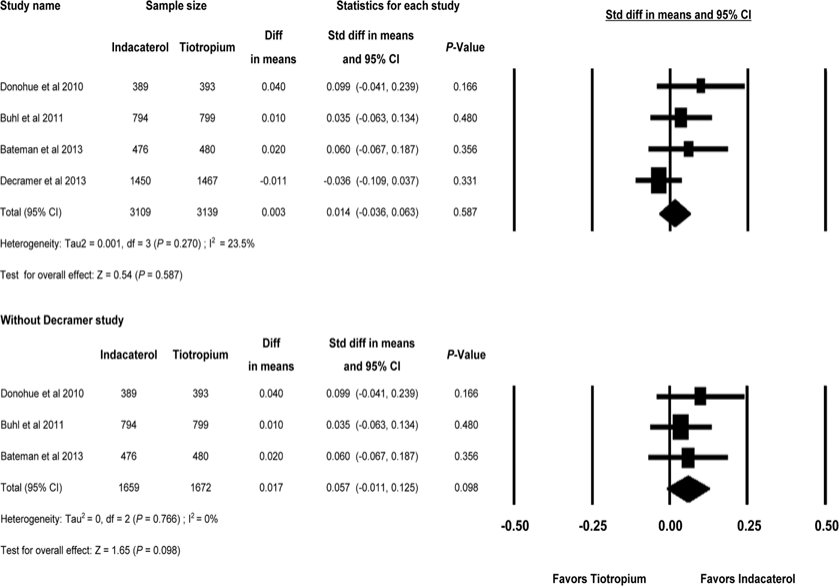
Indacaterol versus Tiotropium on trough FEV_1_ at week 12. FEV_1 =_ forced expiratory volume in 1 second; df = degrees of freedom.

As shown in Fig. 2, the analysis of four studies comparing indacaterol with tiotropium showed no significant differences in trough FEV_1_ at week 12 (MD = 0.003L and SMD = 0.014, 95% CI, -0.036 to 0.063, *P* = 0.587). There was little evidence of statistical heterogeneity (Higgins and Green *I* ^*2*^ = 23.5%, *P* = 0.270, Q = 3.92 for 3 df). When the study of patients with only severe COPD (INVIGORATE) [21] was removed, the heterogeneity among studies dropped to 0%, but trough FEV_1_ at week 12 was not significantly different between indacaterol and tiotropium (*P* = 0.098).

### Secondary outcome

**Fig 3:**
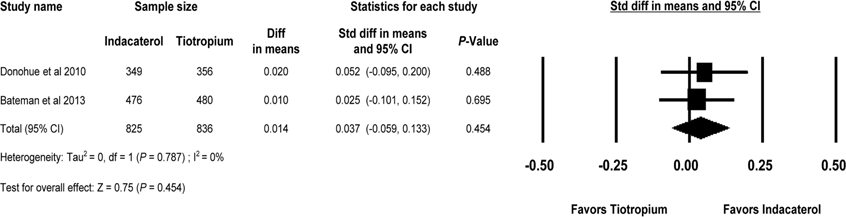
Indacaterol versus Tiotropium on trough FEV_1_ at week 26. FEV_1 =_ forced expiratory volume in 1 second; df = degrees of freedom.

Trough FEV_1_ at week 26 of indacaterol and tiotropium was reported in two studies (INHANCE and SHINE) [18, 20]. There were 825 subjects in the indacaterol 150 μg group and 836 subjects in the tiotropium 18 μg group. The MD of trough FEV_1_ at week 26 between the two groups was 0.014L and the SMD was 0.037, which was not statistically significant (95% CI, -0.059 to 0.133, *P* = 0.454). There was no evidence of statistical heterogeneity (Q = 0.07 for 1 df, *I* ^*2*^ = 0%, *P* = 0.787) (Fig. 3). The test for asymmetry was not assessed because only two RCTs were analyzed.

**Fig 4:**
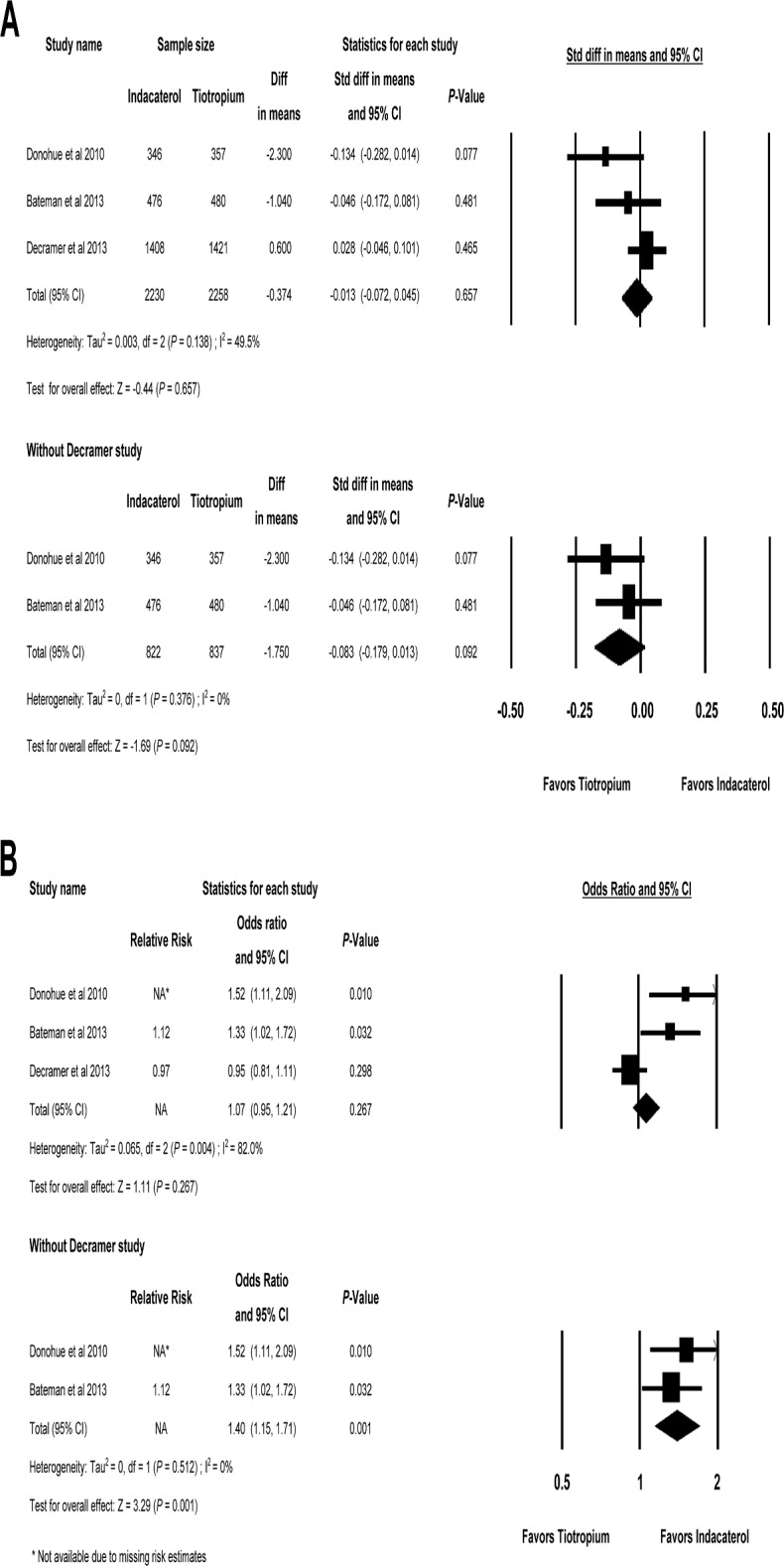
Indacaterol versus Tiotropium on SGRQ at week 26. (A) Pooled standardized difference in means for SGRQ total score at week 26 with 95% CIs of eligible studies comparing indacaterol vs tiotropium. (B) Pooled odds ratio for percentage of patients with MCID (decrease ≥ 4 units) of SGRQ total score at week 26 of eligible studies comparing indacaterol vs tiotropium. SGRQ = St. George`s Respiratory Questionnaire; MCID = minimal clinically important difference; SGRQ = St. George`s Respiratory Questionnaire.

The SGRQ total score at week 26 was reported in three studies (INHANCE, SHINE and INVIGORATE) [18, 20, 21]. As shown in Fig. 4A, the MD was—0.374 and the SMD was-0.013 between indacaterol and tiotropium, which was not significant (95% CI, -0.072 to 0.045, *P* = 0.657) and there was little evidence of heterogeneity (Q = 3.96 for 2 df, I^2^ = 49.5%, *P* = 0.138). When the INVIGORATE study [21] was removed from the analysis, the heterogeneity among studies dropped dramatically (0%), but SGRQ total score at week 26 of indacaterol was not significantly different with that of tiotropium (*P* = 0.092).

Three studies (INHANCE, SHINE and INVIGORATE) presented the decreased SGRQ total score by at least 4 units (MCID) at week 26 [18, 20, 21]. The percentage of patients with MCID in the SGRQ total score was not different between two groups (pooled OR = 1.07, 95% CI, 0.95 to 1.21, *P* = 0.267). The heterogeneity among three studies was substantial by the Higgins and Green test (Q = 11.13 for 2 df, *I* ^*2*^ = 82.0%, *P* = 0.004). Without INVIGORATE study, the heterogeneity became 0% and the percentage of patients with MCID in the SGRQ at week 26 was significantly higher in those using indacaterol than in those receiving tiotropium (pooled OR = 1.40, 95% CI, 1.15 to 1.71, *P* = 0.001) (Fig. 4B).

### Adverse events

As shown in Table 3, the incidence of any adverse events was significantly higher in patients treated with indacaterol than in those treated with tiotropium (58.8% vs. 56.2%, pooled OR = 1.12; 95% CI, 1.01 to 1.23, *P* = 0.028, and RR = 1.04). With regard to adverse events, the incidence of nasopharyngitis was similar between the two once-daily inhaled bronchodilators, while patients using indacaterol experienced cough (6.3% vs. 3.8%, pooled OR = 1.68; 95% CI = 1.34–2.10, *P* < 0.001, and RR = 1.63) and COPD worsening (31.1% vs. 27.8%, pooled OR = 1.18; 95% CI = 1.06–1.32, *P* = 0.003, and RR = 1.12) at higher rates compared to those using tiotropium. When three studies (INHANCE, INTENSITY, and SHINE) [18–20] were used in the meta-analysis, the incidence rates of any adverse events (52.4% vs. 50.2%, pooled OR = 1.09; 95% CI = 0.95–1.26, *P* = 0.206, and RR = 1.04) and COPD worsening (18.4% vs. 16.8%, pooled OR = 1.13; 95% CI = 0.94–1.35, *P* = 0.204, and RR = 1.09) were not significantly different between the two once-daily inhaled bronchodilators. However, the incidence of cough was still higher in patients receiving indacaterol than in those receiving tiotropium (6.2% vs. 4.4% pooled OR = 1.45; 95% CI = 1.07–1.97, *P* = 0.018, and RR = 1.42). In both meta-analyses, there were no significant differences in the rates of serious adverse events or serious cardiovascular adverse events between the two once-daily inhaled bronchodilators.

**Table 3:** Adverse events of Indacaterol vs. Tiotropium monotherapy.

Outcome	No.	No. of studies	Relative Risk	Odds Ratio (95% CI)	I^2^, %	*P* Value
**INHANCE, INTENSITY, SHINE and INVIGORATE studies [18–21]**						
**Any adverse events**	6,819	4	1.04	1.12 (1.01 to 1.23)	0	0.028
**Nasopharyngitis**	6,819	4	1.03	1.04 (0.85 to 1.26)	0	0.720
**Cough**	6,819	4	1.63	1.68 (1.34 to 2.10)	13.03	<0.001
**COPD worsening**	6,819	4	1.12	1.18 (1.06 to 1.32)	11.95	0.003
**Serious adverse events**	6,819	4	1.02	1.03 (0.87 to 1.21)	0	0.748
Serious cardiovascular adverse event* ^**†**^	5,226	3	0.91	0.91 (0.58 to 1.41)	0	0.657
**INHANCE, INTENSITY, and SHINE studies [18–20]** ^‡^						
**Any adverse events**	3,380	3	1.04	1.09 (0.95 to 1.26)	0	0.206
**Nasopharyngitis**	3,380	3	0.92	0.92 (0.70 to 1.21)	0	0.548
**Cough**	3,380	3	1.42	1.45 (1.07 to 1.97)	0	0.018
**COPD worsening**	3,380	3	1.09	1.13 (0.94 to 1.35)	32.84	0.204
**Serious adverse events**	3,380	3	1.01	1.00 (0.73 to 1.38)	17.35	0.982
Serious cardiovascular adverse event* ^**†**^	1,787	2	1.01	1.01 (0.54 to 1.88)	0	0.989

COPD = Chronic obstructive pulmonary disease.

*INTENSITY study was not included in the analysis of serious cardiovascular event.

^†^Serous cardiovascular events of INHANCE study were analyzed with both indacaterol 150ug and 300ug group.

^‡^INVIGORATE study with only severe COPD patients was excluded in subgroup analysis.

## Discussion

This meta-analysis showed that indacaterol (150 μg) was as effective as tiotropium (18 μg) in improving trough FEV_1_ at weeks 12 and 26 among patients with moderate to severe stable COPD. Our data were consistent with those in the meta-analysis reported by Rodrigo et al, [22] which indicated similar efficacy between indacaterol and tiotropium in terms of trough FEV_1_ in moderate to severe COPD patients with the INHANCE and INTENSITY studies [18, 19]. We extended these findings by adding two large RCTs, including the INVIGORATE study conducted only with severe COPD patients [20, 21]. With respect to quality of life, SGRQ total score at week 26 was also similar between the two once-daily inhaled bronchodilators in meta-analysis with INHANCE, SHINE, and INVIGORATE studies [18, 20, 21]. Most importantly, the percentage of patients with MCID in the SGRQ at week 26 showed no difference between those using indacaterol and those using tiotropium, which had substantial heterogeneity in the meta-analysis. As shown in Table 1, one of the four RCTs (the INVIGORATE study) targeted only severe COPD patients. To examine how sensitive our findings are to the one study, we performed another analysis of efficacy, isolating the INVIGORATE study [21]. Similar to the meta-analysis of Rodrigo et al, [22] the results indicated that the percentage of patients with MCID in the SGRQ at week 26 was significantly higher among patients receiving indacaterol than those receiving tiotropium with the absence of heterogeneity in meta-analysis of the INHANCE and SHINE studies [18, 20]. However, the result needs to be interpreted with caution, because MCID differences were not derived on the basis of differences between two once-daily inhaled bronchodilators; indacaterol (150 μg) and tiotropium (18 μg).

In addition to efficacy, adverse events are key factors in choosing and maintaining a particular bronchodilator. The present meta-analysis indicated that patients receiving indacaterol had significantly higher rates of any adverse event, COPD worsening, and cough compared to those treated with tiotropium, while the rates of nasopharyngitis, serious cardiovascular adverse events, such as cardiac failure and myocardial ischemic disease, and serious adverse events did not differ between indacaterol and tiotropium. We conducted a meta-analysis of the adverse events as well as efficacy using three RCT studies (INHANCE, INTENSITY, and SHINE) with moderate and severe COPD patients and excluding the INVIGORATE study. The results showed no statistically significant differences in rates of any adverse events or COPD worsening between patients treated with indacaterol and those treated with tiotropium [18–20]. In these three RCTs, the mean FEV_1_% predicted was 54–56%, indicating that more than half of the population in each study (≥ 60% in INTENSITY and SHINE) consisted of moderate COPD patients [19, 20]. On the other hand, only severe COPD patients were enrolled in the INVIGORATE study [21] and the number of subjects (*n* = 3439) was similar to the total number of subjects in the other three RCTs (*n* = 3380). It suggests that disease severity may explain these differences in adverse events and COPD worsening between meta-analyses. Besides the severity of COPD and the number of participants, multiple other factors might have influenced these different results. Given that COPD exacerbations were included in COPD worsening, the durations of the three RCTs (INHANCE, INTENSITY, and SHINE) [18–20] were relatively short (12–26 weeks) to evaluate COPD worsening compared to the INVIGORATE study (52 weeks) [21].

Regardless of whether we performed the analysis with three RCTs (INHANCE, INTENSITY, and SHINE) [18–20] or four RCTs (with addition of the INVIGORATE study) [18–21], patients who received indacaterol showed a higher rate of cough than those treated with tiotropium. Cough is the most commonly reported adverse effect associated with the use of indacaterol. Indacaterol was initially developed as the maleate salt, which was associated with a cough occurring post-inhalation according to the manufacturer’s report. The post-inhalation cough occurred within 15 s after inhalation and lasted for around 6 s, and was not associated with post-inhalation bronchospasm or any negative effects on safety or efficacy [23].

The review was performed according to the methodological criteria suggested by scientific guidelines [24]. Inclusion criteria were clearly defined and the risk of bias was formally assessed. As with all meta-analyses, there is a potential bias in analyzing published studies, which are more likely to have positive results. We investigated this possibility using Egger`s regression test for asymmetry, and the funnel-plot based Trim and Fill method. The Egger’s regression test suggested a significant asymmetry, but the Trim and Fill adjusted test did not reverse the fixed effects model result, confirming that there was no significant difference in the primary outcome. However, as we analyzed only four studies, statistical power was expected to be low [16, 17].

In summary, this study suggests that clinical effects on lung function and quality of life were not different between the two once-daily inhaled bronchodilators; indacaterol (150 μg) and tiotropium (18 μg), with acceptable safety profiles. Cough was the most common adverse effect associated with indacaterol, and COPD worsening occurred more frequently in patients treated with indacaterol than in those treated with tiotropium, particularly among patients with severe COPD.

## Supporting Information

S1 PRISMA Checklist (DOC)

S1 Table Search terms based on databases. (DOCX)
