# Supplementary material for: Comparison of Clinical Efficacy and Safety between Indacaterol and Tiotropium in COPD: Meta-Analysis of Randomized Controlled Trials
Source: PLoS One. 2015 Mar 23;10(3):e0119948. doi: 10.1371/journal.pone.0119948 (PMC4370711; doi:10.1371/journal.pone.0119948)
Supplement: S1 Table — (DOCX) [file pone.0119948.s002.docx]

**Table S1** Search terms based on databases

| **Mesh** | **Emtree** | **Text** |
| --- | --- | --- |
| Lung Diseases, Obstructive | emphysema | Chronic obstructive pulmonary disease |
| Pulmonary Disease, Chronic Obstructive | chronic bronchitis | Emphysema |
| Pulmonary Emphysema | obstructive lung disease | Chronic bronchitis |
| indacaterol | obstructive pulmonary disease | Chronic obstructive lung disease |
| Indans | copd | Obstructive pulmonary disease |
| Adrenergic beta-2 Receptor Agonists | lung emphysema | COPD |
| Quinolones | chronic obstructive lung disease | Arcapta |
|  | indacaterol | hirobriz breezhaler |
|  |  | indacaterol |
|  |  | onbrez |
|  |  | oslif breezhaler |
|  |  | qab 149 |
|  |  | long-acting bronchodilators |
|  |  | LABA |
|  |  | arcapta neohaler |
